# Study on the growth of Al-doped ZnO thin films with (112̄0) and (0002) preferential orientations and their thermoelectric characteristics

**DOI:** 10.1039/c7ra12485f

**Published:** 2018-02-06

**Authors:** Jing-Ting Luo, Ao-Jie Quan, Zhuang-Hao Zheng, Guang-Xing Liang, Fu Li, Ai-Hua Zhong, Hong-Li Ma, Xiang-Hua Zhang, Ping Fan

**Affiliations:** Institute of Thin Film Physics and Applications, Shenzhen Key Laboratory of Advanced Thin Films and Applications, College of Physics and Energy, Shenzhen University 518060 China fanping308@126.com zhongah@szu.edu.cn; Laboratory of Glasses and Ceramics, Institute of Chemical Science UMR CNRS 6226, University of Rennes 1 Rennes 35042 France

## Abstract

In this work, using a conventional magnetron sputtering system, Al-doped ZnO (AZO) films with (112̄0) and (0002) preferential orientations were grown on *r*-sapphire and *a*-sapphire substrates, respectively. The effect of substrate and deposition temperature on the growth of AZO films and their preferential orientations were investigated. The crystallographic characteristics of AZO films were characterized by X-ray diffraction (XRD). The surface morphology of AZO films was studied by scanning electron microscopy (SEM) and atomic force microscopy (AFM). It is found that the lattice mismatch between AZO and substrate determines the growth of AZO films and their preferential orientations. The thermoelectric properties are strongly dependent on the crystal grain shape and the grain boundaries induced by the preferred orientation. The highly connected and elongated grains lead to high thermoelectric properties. The in-plane anisotropy performances of thermoelectric characteristics were found in the (112̄0) preferential oriented ZnO films. The in-plane power factor of the (112̄0) preferential oriented ZnO films in the [0001] direction was more than 1.5 × 10^−3^ W m^−1^ K^−2^ at 573 K, which is larger than that of the (0002) preferential oriented ZnO films.

## Abstract 2

In this work, using a conventional magnetron sputtering system, Al-doped ZnO (AZO) films with (112̄0) and (0002) preferential orientations were grown on *r*-sapphire and *a*-sapphire substrates, respectively.

## Graphical abstract

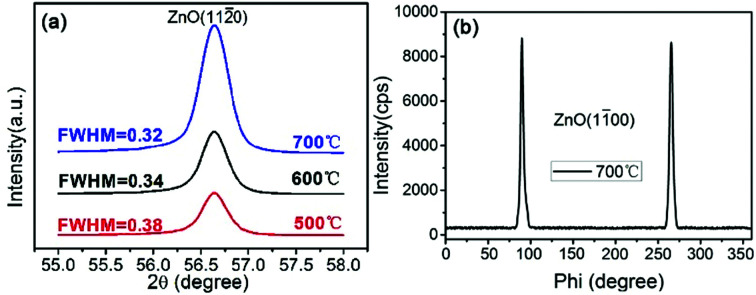

## 1. Introduction

With the increase in the energy crisis and environmental pollution, sustainable energy technology has attracted more and more attention.^1–3^ Thermoelectric generators can convert heat directly into electricity without using steam and mechanical processes, and have become a hot research field throughout the world.^4,5^ Thermoelectric application requires thermoelectric materials possessing a large power factor (PF = *σS*^2^, where *σ* is the electrical conductivity, *S* is the Seebeck coefficient) and low thermal conductivity (*κ*).^6^ ZnO has attracted much attention as an alternative for n-type thermoelectric oxide materials due to its high PF, high chemical and thermal stability at elevated temperatures, non-toxicity and low-cost production from abundant raw materials.^7–11^ As for thermoelectric application, the electrical resistivity of undoped ZnO materials is too high.^12^ Fortunately, its electrical resistivity can be significantly reduced *via* doping. Therefore, various dopants were introduced into ZnO to improve its thermoelectric properties.^3,8,13–15^ Among these works, it is found that Al-doped ZnO (AZO) is one of the best thermoelectric materials for high-temperature thermoelectric application.^12,16^ Thermoelectric materials in low-dimensional are promising to possess better thermoelectric performance than their bulk materials.^1,17,18^ A promising approach to achieve the low-dimensional thermoelectric materials is to deposit thin films, which are suitable for small-scale thermoelectric applications.^19^ This is why so many research group focused on the investigation on the growth of high quality ZnO thin films for thermoelectric applications. ZnO thin film, a versatile multifunctional material,^20–22^ which has been widely used in solar cells,^23^ surface acoustic waves,^24–27^ dilute magnetic semiconductors,^28^ resistive memory,^29^*etc.* Generally, ZnO is known to readily exhibit (0002)-preferred with the *c*-axis perpendicular to the substrate due to the lower surface free energy for the (0002) plane on single-crystalline substrates and even on amorphous substrates such as glass, and polymer substrate^18,30^_. Unusual, some researchers reported theoretical and experimental works concerning other crystalline planes such as (112̄0)- and (1000)-oriented ZnO films^31–36^ and ZnO films with different crystalline orientations possess different electronic, optical and acoustic properties. The thermoelectric anisotropic properties between *ab*-plane and along the *c*-axis of ZnO based films or ceramics with the *c*-axis perpendicular to the substrate has been reported. However, to our knowledge, the thermoelectric properties of (112̄0)-oriented ZnO films have been rarely reported and their thermoelectric characteristics of ZnO films with different crystalline orientations have not been reported in detail yet. Generally, specific deposition techniques are used to deposit ZnO films with good quality, for example pulsed laser deposition technique.^37,38^ In this work, we use a simple magnetron sputtering technology to deposit AZO films. The growth of AZO thin films with (112̄0) and (0002) preferential orientations and their thermoelectric characteristics of ZnO films with different grain shape and size was investigated. In general, the sapphire is the most commonly used substrate for growing ZnO films with different preferred-orientations. Furthermore, sapphire has low thermal conductivity, which is good for thermoelectric application. Therefore, *r*-sapphire and *a*-sapphire substrates were used to grow AZO films with (112̄0) and (0002) preferential orientations respectively. It is found that AZO films deposited on *r*-sapphire and *a*-sapphire substrates exhibit different grain size and orientation which, in turn, leads to the difference in thermoelectric performance.

## 2. Experimental details

AZO thin films were deposited by radio frequency magnetron sputtering using a ZnO : Al (with 2 at% Al) ceramic target (99.995% pure). The single crystalline *r*-sapphire and *a*-sapphire substrates was used. The substrate was cut into rectangular pieces and ultrasonically cleaned in acetone, alcohol and deionized water for 8 minutes, respectively. The sputtering chamber was pumped to a base pressure of 1.0 × 10^−4^ Pa, and then a mixture of highly pure argon (99.999%) and oxygen (99.999%) gases was introduced into the chamber. The working pressure was kept at 0.5 Pa with 40 sccm of Ar and 5 sccm of O_2_. Prior to deposition, a 15 min pre-sputtering process was performed to remove native oxides and contaminants on the surfaces of the ZnO : Al target. The distance between the target and substrates was fixed at 70 mm. The sputtering power was kept at 100 watts and the deposition rate was about 20 nm min^−1^. According to our previous work, the stress of the thin films would be minimum when the thickness of AZO films is around 600 nm. Therefore, the deposition time was fixed at 30 minutes. After deposition, the thicknesses of the AZO films were measured using a stylus profilometer and all the ZnO films have similar thickness (∼600 nm).

The structure of the prepared AZO films was studied by X-ray diffraction (XRD) using Cu-K_α_ (*λ*_α_ = 0.15406 nm) radiation with the conventional *θ*–2*θ* mode. XRD Phi-scans was employed to investigate the epitaxial relationship between ZnO films and the substrate diffraction. The surface morphology of the deposited AZO films were examined by scanning electron microscope (SEM) and atomic force microscopy (AFM). The room temperature carrier concentrations and mobility of rectangular AZO thin film samples along the length and width direction, respectively, were evaluated using Hall measurement system in a four-probe van der Pauw configuration. The electrical conductivity of the AZO films were measured by the home-made four-point-probe technique. The four point measurements are co-linear and the conductivities of all the rectangular samples were measured along length and width, respectively. Seebeck coefficient was measured with the temperature gradient method (Δ*T* = 20 K) using a SDFP-1 measurement system and the Seebeck coefficient of all the rectangular samples were measured along length and width, respectively. All the measurement temperature was changed from room temperature to 573 K.

## 3. Results and discussion

**Fig. 1:**
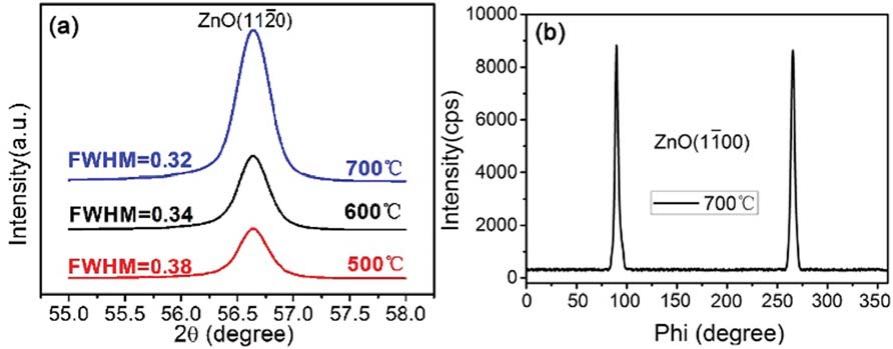
(a) *θ*–2*θ* XRD patterns of AZO films deposited on *r*-sapphire substrate at the substrate temperature of 500 °C, 600 °C and 700 °C. (b) The phi-scans through off normal ZnO (11̄00) reflections of AZO films deposited at 700 °C.

The crystal structures of all the deposited AZO films were investigated by XRD. Fig. 1(a) shows the XRD patterns of AZO films deposited on *r*-plane sapphire substrates with the substrate temperatures of 500 °C, 600 °C and 700 °C. The reflections observed corresponding to ZnO plane centered at 56.6°, with two additional substrate reflections arising from the sapphire (22̄04) and (33̄06) plane. No other peaks can be seen in the patterns under the detection limit of XRD, indicating that the deposited ZnO films have preferred (112̄0) orientation with *c*-axis parallel to the substrate. From these reflections, it is suggested that the out of plane epitaxial relationship between ZnO films and the sapphire substrates is (112̄0)‖(22̄04). And the epitaxial relationship does not change with the increase of substrate temperature. However, the peak intensity of ZnO (112̄0) increases and the full-width at half-maximum (FWHM) value of ZnO (112̄0) decreases a little with the increase of substrate temperature, indicating that ZnO film has relative stronger (112̄0) texture and better crystalline at the elevated temperature.

Azimuthal scans (phi-scans) were performed to investigate the in-plane epitaxial relationship between ZnO film and *r*-sapphire substrate. Since ZnO films deposited at the substrate temperature of 700 °C has the best crystalline, Fig. 1(b) shows the phi-scans through off normal ZnO (11̄00) reflections of AZO films deposited at 700 °C. In the range of 0–360°, only two diffraction peaks can be seen in the pattern and they are spacing at ∼180°, indicating that ZnO films in *a*-plane shows two-fold symmetry. Based on the phi-scans of *r*-sapphire, we conclude that the in-plane epitaxial relationship between ZnO film and substrate is [0001]_ZnO_‖[1̄101]_Al_2_O_3__ and [1̄100]_ZnO_‖[1̄1̄20]_Al_2_O_3__, which is coincident to the previous literatures.^34,39^

**Fig. 2:**
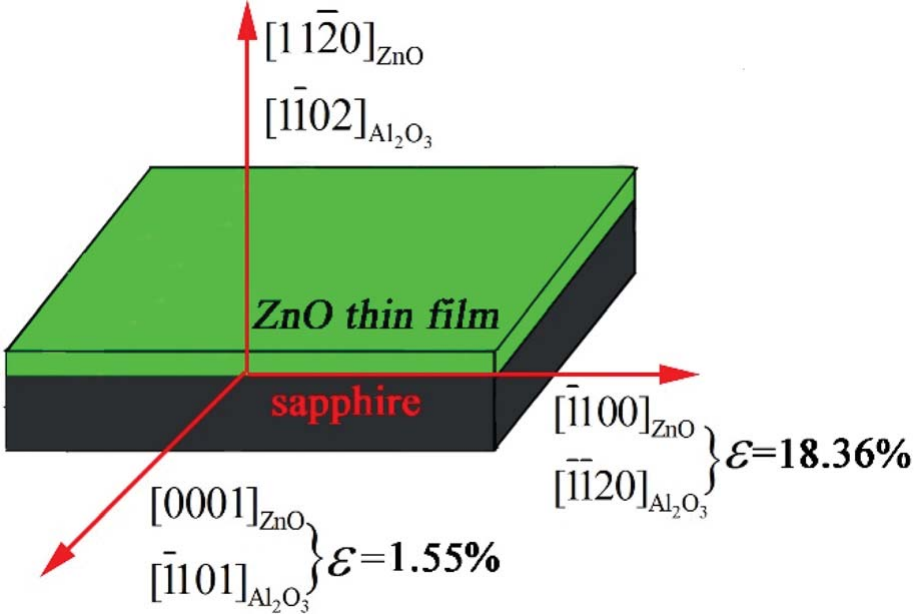
The schematic illustration of the in-plane epitaxial relationship between ZnO film and *r*-sapphire substrate.

Fig. 2 illustrates the in-plane epitaxial relationship between ZnO film and the sapphire substrate. The calculated lattice mismatch along the two different directions in-plane is marked in the diagram. Along the *c*-axis of ZnO (ZnO [0001] direction), the in-plane lattice parameters of ZnO [0001] and sapphire [1̄101] are 5.2069 and 5.1272 Å, respectively,^34^ and the mismatch of ZnO thin film in-plane along [0001] direction is 1.55% ((5.2069−5.1272)/5.1272). For the direction along ZnO [1̄100], the lattice parameters of ZnO [1̄100] and sapphire [1̄1̄20] are 5.629 and 4.756 Å respectively,^34^ and the mismatch of ZnO thin film in-plane along [1̄100] direction is 18.36% ((5.629−4.756)/4.756). It can be seen that ZnO thin film has structure anisotropy in-plane and the mismatch along the [0001] direction is much smaller than along [1̄100] direction. The mismatch along [1̄100] direction can be made up by the two alternate domains of ZnO thin films, namely five ZnO (11̄00) planes matches with six sapphire (112̄0) planes, as well as six ZnO (11̄00) planes matches with seven sapphire (112̄0) planes.

**Fig. 3:**
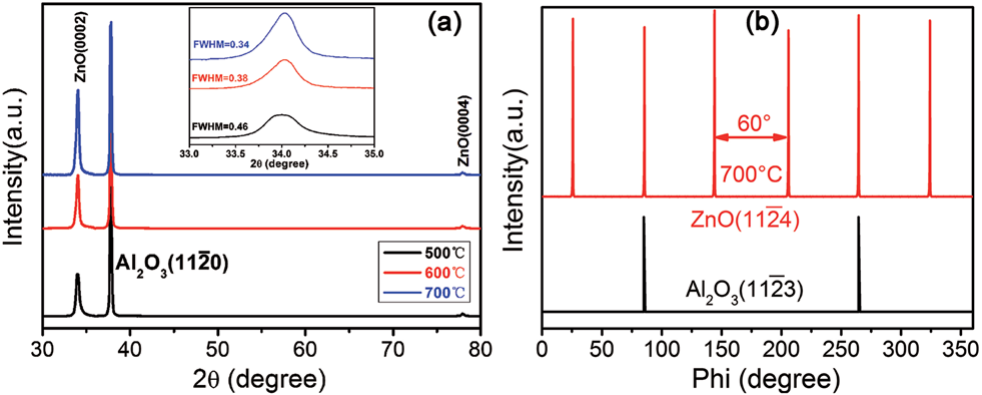
(a) *θ*–2*θ* XRD patterns of AZO films deposited on *a*-sapphire substrate at the substrate temperature of 500 °C, 600 °C and 700 °C. (b) Phi-scans of ZnO films deposited on *a*-sapphire substrate at 700 °C.

The XRD patterns of the AZO thin films grown on *a*-sapphire at 500 °C, 600 °C and 700 °C showed in Fig. 3(a). The two diffraction peaks centered at ∼34.4° and ∼72.5° are characteristic of the wurtzite structure of ZnO, corresponding to the reflections of ZnO (0002) and (0004) planes, respectively.^33^ There is another peak centered at ∼37.7° arising from the (112̄0) planes of the *a*-sapphire substrate. No other peaks exhibited in the patterns under the detection limit of XRD, indicating that the deposited ZnO films have preferred (0002) orientation with *c*-axis perpendicular to the substrate. The peak intensity of ZnO (0002) increases and the FWHM value of ZnO (0002) decreases from 0.46° to 0.34° with the substrate temperature increase from 500 °C to 700 °C, indicating that ZnO film has better crystalline with the increase of substrate temperature. In addition, the microstructure and crystalline preferred orientation do not change with increasing in substrate temperature. Therefore, we chose ZnO films with better crystallinity deposited at 700 °C to investigate the epitaxial relationship between ZnO film and sapphire using Phi-scans. Fig. 3(b) shows the phi-scans through off normal ZnO (112̄4) reflections of ZnO films deposited at 700 °C together with the phi-scan through *a*-sapphire (112̄3) plane. The figure shows six-fold symmetry with about 60° intervals, indicating a good hexagonal symmetry. Two peak positions of ZnO (112̄4) match good with that of sapphire substrate with (112̄3) reflections, yielding an in-plane epitaxial relationship of [1̄100]_ZnO_‖[1̄100]_Al_2_O_3__.

It is reported that the different substrate caused different grain size and shape of AZO thin films,^15^ which result in different thermoelectric performance. The grain size of AZO films can be calculated using Scherrer equation:

1 *D* = *kλ*/(*β* cos *θ*)

where *k* is a constant, *λ* is the wavelength of the X-ray, here we used Cu-K_α_ (*λ*_α_ = 0.15406 nm), *β* is the FWHM, *θ* is the diffraction angle. Based on the above formula, the grain size of AZO films deposited on different substrates at 500 °C, 600 °C and 700 °C are shown in Table 1. As can be seen from Table 1, with the increase of the deposition temperature, the FWHM decrease and the grain size increase, this is because the higher deposition temperature provide more sufficient energy for AZO grains growth, and the recrystallization phenomenon of AZO grains become significant and the crystallinity would be better.

**Table 1:** The grain size of AZO films deposited on different substrates at 500 °C, 600 °C and 700 °C

AZO films	FWHM (°)	*D* (nm)
AZO/*r*-sapphire at 500 °C	0.38	24.9
AZO/*r*-sapphire at 600 °C	0.34	27.8
AZO/*r*-sapphire at 700 °C	0.32	29.5
AZO/*a*-sapphire at 500 °C	0.46	17.9
AZO/*a*-sapphire at 600 °C	0.38	21.6
AZO/*a*-sapphire at 700 °C	0.34	24.2

**Fig. 4:**
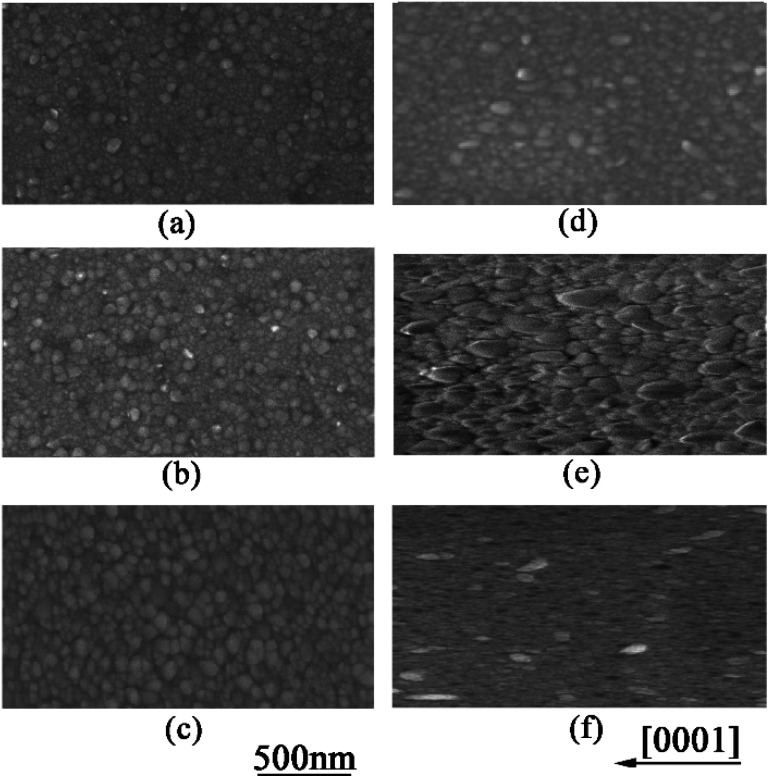
SEM image of AZO thin film deposited on *a*-sapphire at 500, 600, and 700 °C (a)–(c), and on *r*-sapphire at 500, 600 and 700 °C (d)–(f).

Furthermore, SEM and AFM were used to characterize the surface morphology of AZO films. Fig. 4(a)–(c) show the top-view SEM images of AZO films deposited on *a*-sapphire at 500 °C, 600 °C and 700 °C, respectively. Fig. 4(a)–(c) show nearly rounded grains morphology of AZO deposited on *a*-sapphire and AZO films deposited at 700 °C has denser and uniform grains than that deposited at 500 °C and 600 °C. Fig. 4(d)–(f) show the top-view SEM images of AZO films deposited on *r*-sapphire at 500 °C, 600 °C and 700 °C, respectively. It shows elongated grains morphology commonly observed on ZnO film with (112̄0) preferred orientation.^40,41^ The elongated direction is parallel to [0001] usually seen on ZnO film with (112̄0) preferred orientation.^42^

**Fig. 5:**
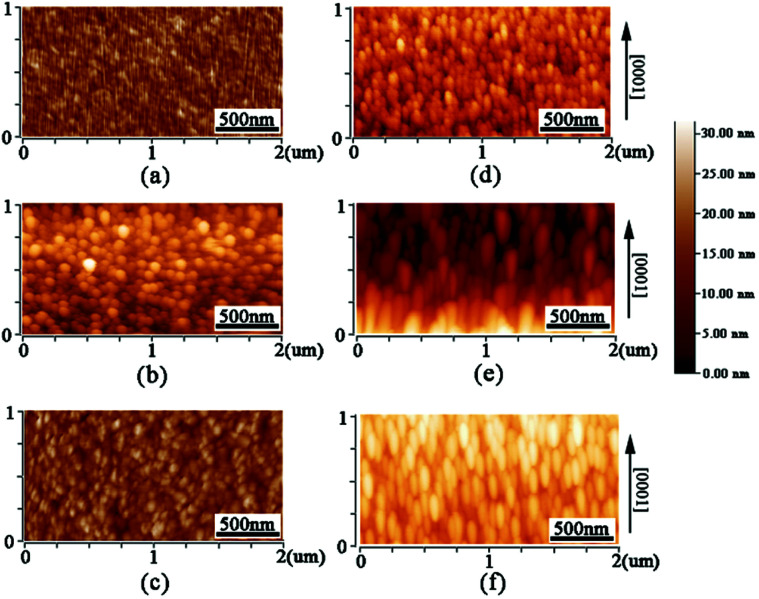
AFM images of AZO films deposited on *a*-sapphire at 500, 600 and 700 °C (a)–(c) and AZO films deposited on *r*-sapphire at 500, 600 and 700 °C (d)–(f).

We also used AFM to confirm the influence of substrate on the grain shape of AZO films. Fig. 5 (a)–(c) show the AFM images of AZO films deposited on *a*-sapphire at 500, 600 and 700 °C, which (d)–(f) show the AFM images of AZO films deposited on *r*-sapphire at 500, 600 and 700 °C, respectively. AZO films deposited on *a*-sapphire exhibits rounded and uniform grains as shown in Fig. 5(a)–(c) and they seem isotropic in all directions. AZO films deposited on *r*-sapphire show the elongated grains with different sizes as shown in Fig. 5(d)–(f). AZO films show stretched morphology in [0001] direction, illustrating anisotropic in-plane ZnO thin film. It is suggested that the stretch direction is along [0001] direction since the required growth momentum along [0001] direction is minimum in AZO (112̄0) plane. With the increase of substrate temperature, the stretched morphology is more and more obvious. This is because the higher temperature provide more sufficient energy for the growth of AZO grains. The thin film morphology as revealed by AFM showed good agreement with SEM results. Therefore, one can conclude that substrate strongly affects the grain shape and size of AZO films.

**Fig. 6:**
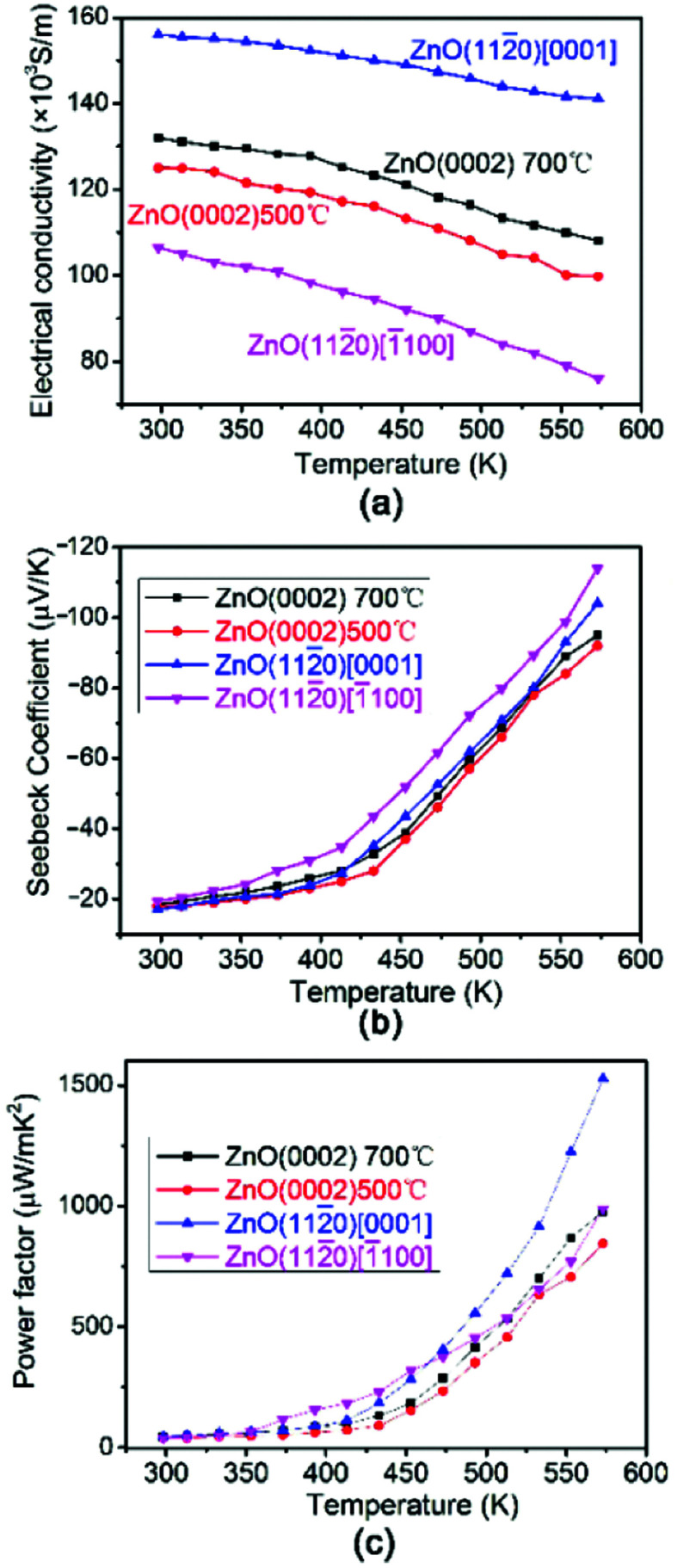
The thermoelectric properties of AZO thin films with different orientation as a function of the temperature. (a) Electrical conductivity (b) Seebeck coefficient and (c) power factor.

The electrical conductivity *σ* of AZO thin films with different orientation depends on the testing temperature is shown in Fig. 6(a). The *σ* values of all the films decrease slightly with the increase of measuring temperature, indicating the metallic electrical conductivity behaviour. The carrier concentrations of AZO films shown in Table 2 are around 22–24 × 10^20^ cm^−3^, which are similar to that of metal conductor. The prepared AZO films are n-type semiconductor and the carrier concentration is so high that the Fermi energy is approaching or entering the conductive band. Therefore, AZO films become n-type degenerate semiconductor. It is well known that the degenerate semiconductor exhibit metallic electrical conductivity behavior. Similarly, the deposited AZO thin films exhibit metallic electrical conductivity behavior. The conductivity of AZO (112̄0) thin films along [0001] is larger than all the other films and AZO (112̄0) thin films show anisotropic electrical conductivity in plane along [0001] and [1̄100] direction. All the AZO films deposited at 700 °C shows similar carrier concentrations regardless of preferred orientation, however, AZO films deposited at 700 °C shows larger carrier concentrations than that of AZO films deposited at 500 °C as shown in Table 2. AZO (112̄0) thin film along [0001] direction possesses large elongated grains parallel to the substrate, which can be used as the efficient carrier pathway. Furthermore, it has less grain boundaries along [0001] direction, and the electron can transport easier in AZO (112̄0) thin films along [0001] direction. Therefore, AZO (112̄0) thin films along [0001] direction exhibits largest carrier mobility as shown in Table 2, which results in high conductivity. AZO (0002) thin films have columnar structure with large grains perpendicular to the substrate, making more grain boundaries in *ab* plane. The larger amount of grain boundaries in-plane induces stronger scattering of electrons. Therefore, the carrier mobility as shown in Table 2 in AZO (0002) thin films are smaller than that of AZO (112̄0) thin films along [0001] direction and the conductivity of AZO (0002) thin films is smaller. However, with the increase of substrate temperature, the carrier concentration increases and the crystalline improves with denser and uniform grain (see Fig. 4(b) and (c)), which makes the electron transport easier. That is why the conductivity of AZO (0002) thin film deposited at 700 °C is larger than that deposited at 500 °C. It is reported that AZO (112̄0) thin film along [1̄100] direction is the least stable and tends to form long groove, which make the electrons transport more difficult.^35^ Therefore, although it has similar carrier concentration, the carrier mobility is smaller as shown in Table 2. That is why the AZO (112̄0) thin film along [1̄100] direction has the lowest conductivity.

**Table 2:** The carrier concentration and mobility of AZO films with different orientation measured at room temperature

AZO films	Carrier concentration (×10^20^ cm^−3^)	Mobility (cm^2^ V^−1^ s^−1^)
AZO (0002) 700 °C	23.4	15.6
AZO (0002) 500 °C	22.8	15.1
AZO (112̄0)[0001]	23.7	18.2
AZO (112̄0)[1̄100]	23.1	12.3

In short, we believe that the different grain shapes and sizes induced by the preferred orientation results in different conductivities. The substrate effects on the conductivity of AZO thin films were also reported by Saini *et al.*^43^ They found that AZO thin films deposited on Al_2_O_3_ substrate exhibited c-axis oriented and larger electrical conductivity than deposited on SrTiO_3_ and fused silica.

Fig. 6(b) shows the testing temperature dependence of the Seebeck coefficient of AZO (0002) thin films deposited at different temperature and AZO (112̄0) thin films along different direction. As can be seen from the figure, the negative *S* indicates that all of the AZO films are n-type semiconductor thermoelectric materials. This is expected since Al dissolved in the ZnO crystal lattice acting as donor, which will create extra electrons. In general, Seebeck coefficient *S* can be expressed as following:^19^2
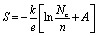
where *k* is Boltzmann constant, *e* is the electron charge, *N*_c_ is the effective density of states, *n* denotes carrier concentration and *A* is a transport constant. One can find from the formula that when the carrier concentration decrease the Seebeck coefficient *S* increase. As AZO (112̄0) thin films along [1̄100] direction have smaller carrier concentration and mobility, the absolute value of *S* of AZO (112̄0) thin films along [1̄100] direction is larger than others. The *S* of other samples does not exhibit too much difference as shown in Fig. 6(b).

From the values of Seebeck coefficient and electrical conductivity, we have calculated the PF of thin films depends on the testing temperature as shown in Fig. 6(c). The PF of all the AZO films increases with increasing temperature. The power factor of (112̄0) preferential oriented AZO film in [0001] direction is more than 1.5 × 10^−3^ W m^−1^ K^−2^ at 573 K because of the large electrical conductivity, which is larger than all the other preferential oriented ZnO films. The PF in our work is comparable to the previously reported values.^12,44,45^ The PF of AZO (0002) thin film deposited at 700 °C is slightly larger than that of AZO (0002) thin film deposited at 500 °C, indicating that PF depends slightly on orientation degree in AZO (0002) thin films. Saini *et al.*^43^ reported that AZO thin film deposited on fused silica substrate showed as large as −200 μV K^−1^, however, the small *σ* limited its PF. Fortunately, the formation of the seed layer lead to the significant decrease of thermal conductivity, which improved the thermoelectric figure.

## 4. Conclusions

AZO thin films with (112̄0) and (0002) preferential orientations were respectively grown on *r*-sapphire and *a*-sapphire substrates using a conventional magnetron sputtering method. AZO film shows relative stronger preferred orientation and better crystallinity with the increase of substrate temperature. The electrical conductivity is strongly depended on the crystal grain shape and the grain boundaries induced by preferred orientation. Due to the elongated grains and less grain boundaries, the electrical conductivity of (112̄0) oriented ZnO films along [0001] direction is much larger than that of all other preferential oriented ZnO films. The in-plane PF of (112̄0) preferential oriented AZO films in [0001] direction was more than 1.5 × 10^−3^ W m^−1^ K^−2^ at 573 K, which are larger than that of all the other preferential oriented AZO films.

## Conflicts of interest

There are no conflicts to declare.

## Supplementary Material
